# Sustainable Development of ZnO Nanostructure Doping with Water Hyacinth-Derived Activated Carbon for Visible-Light Photocatalysis

**DOI:** 10.3390/toxics12030165

**Published:** 2024-02-21

**Authors:** Sucheewan Krobthong, Tipawan Rungsawang, Naphatson Khaodara, Napat Kaewtrakulchai, Kanit Manatura, Khewika Sukiam, Donchida Wathinputthiporn, Sawitree Wongrerkdee, Chatdanai Boonruang, Sutthipoj Wongrerkdee

**Affiliations:** 1 Department of Physical and Material Sciences, Faculty of Liberal Arts and Science, Kasetsart University, Kamphaeng Saen Campus, Nakhon Pathom 73140, Thailand; 2 Kasetsart Agricultural and Agro-Industrial Product Improvement Institute, Kasetsart University, Bangkok 10900, Thailand; 3 Department of Mechanical Engineering, Faculty of Engineering at Kamphaeng Saen, Kasetsart University, Kamphaeng Saen Campus, Nakhon Pathom 73140, Thailand; 4 Department of Tourism and Aviation Business, Faculty of Hospitality Industry, Kasetsart University, Kamphaeng Saen Campus, Nakhon Pathom 73140, Thailand; 5 Department of Agricultural Extension and Communication, Faculty of Agriculture at Kamphaeng Saen, Kasetsart University, Kamphaeng Saen Campus, Nakhon Pathom 73140, Thailand; 6 Faculty of Engineering, Rajamangala University of Technology Lanna Tak, Tak 63000, Thailand; 7 Department of Physics and Materials Science, Faculty of Science, Chiang Mai University, Chiang Mai 50200, Thailand; 8 Center of Excellence in Materials Science and Technology, Chiang Mai University, Chiang Mai 50200, Thailand

**Keywords:** water hyacinth, activated carbon, ZnO, photocatalysis

## Abstract

Water hyacinth (Wh) is an aquatic weed considered a nuisance in agricultural and fishing activities. Therefore, this study proposed repurposing this plant into activated carbon (AC). First, the ZnO-AC was precipitated and applied as a photocatalyst for degrading methylene blue. The preliminary photocatalytic test under UV irradiation identified the optimum ZnO-AC photocatalyst to degrade methylene blue (MB). The ZnO-AC photocatalyst recorded the highest degradation rate constant of 11.49 × 10^−3^ min^−1^, which was almost two-fold higher than that of ZnO (5.55 × 10^−3^ min^−1^). Furthermore, photocatalytic degradation of MB and carbaryl under sunlight irradiation by ZnO-AC demonstrated degradation rate constants of 74.46 × 10^−3^ min^−1^ and 8.43 × 10^−3^ min^−1^, respectively. To investigate the properties of ZnO-AC, several techniques were performed. ZnO-AC and ZnO exhibited similar results in morphology, crystalline structure, and Raman characteristics. However, ZnO-AC presented smaller pore diameters than those of ZnO, which enlarged pore surface area, and the presence of carbon-related groups implied the presence of AC on ZnO-AC surfaces. This can be attributed to the presence of AC on the ZnO surface, increasing the capture of surrounding toxic molecules and elevating the reaction density. This mechanism is attributed to promoting the degradation of toxic molecules. Therefore, using Wh as a carbon source for the transformation of AC can alternatively solve the problems of aquatic weed management and carbon storage strategies, and the application of AC in ZnO-AC photocatalysts can enhance photocatalysis.

## 1. Introduction

Numerous methodologies have been developed to manage wastewater contaminants. Photocatalysis is one of the most promising approaches due to its efficacy, non-selectivity, and efficiency, which enable repeated usage of the photocatalyst [1,2,3]. Furthermore, photocatalysis facilitates the removal of various contaminants via mineralization or decomposition of intricate pollutants, such as landfill leachate, into simpler components like water, carbon dioxide, and inorganic ions. Consequently, photocatalysis has been proven to be effective for mineralizing complex contaminants, offering a viable alternative technology capable of degrading and eliminating complex pollutants. Notably, researchers have greatly utilized this method, particularly in developing nano-photocatalysts. Nanostructured photocatalysts are superior to their conventional counterparts as they offer larger surface areas and a higher prevalence of lattice defects [4]. These features provide additional reactive sites and excellent light absorption properties, thus improving photocatalytic efficiency. Several conventional techniques for fabricating nanoscale photocatalysts include hydrothermal, solvothermal, sol-gel, and precipitation methods have been investigated [5,6,7]. These approaches enable meticulous regulation of the photocatalyst’s particle size and morphology.

Metal oxide semiconductors such as TiO_2_, SnO_2_, and ZnO have been investigated in recent studies. Reports have highlighted ZnO as a crucial semiconducting photocatalyst due to its photosensitivity, stability, low toxicity, availability, excellent electron mobility, cost-effectiveness, and flexibility for use in several synthesis techniques [4,8]. However, the rapid recombination of electron–hole pairs in its structure limits its photocatalytic application [9]. Therefore, reducing recombination and inducing carrier migration in photocatalysts may offer a solution to this issue. These improvements could be achieved by fabricating composite structures, such as fabricating ZnO/Cu-DPA nanocomposites with different levels of Cu-DPA [10]. Photocatalytic activities of nanocomposites are assessed based on the ability to degrade methylene blue (MB) under visible-light irradiation. An earlier study reported that the maximum degradation efficiency and degradation rate of the optimum ZnO/Cu-DPA nanocomposite (78.5%, 23 × 10^−3^ min^−1^) were higher than those for ZnO (71.8%, 16 × 10^−3^ min^−1^). This enhancement was caused by the formation of a p–n heterojunction within the composite system. In the ZnO/Cu-DPA nanocomposite, Cu_2_O is responsible for the photocatalytic activity due to its strong absorption of visible light, promoting optimal separation of electron–hole pairs. Moreover, heterostructure formation with ZnO prevented the recombination of photogenerated charge carriers and confined the photo-excited charges at intrinsic defect sites within the ZnO structure. The ZnO/Cu-DPA nanocomposite facilitates the conversion of dissolved oxygen (O_2_) into hydroxyl (^•^OH) radicals, suggesting that ^•^OH is the primary active radical involved in the photocatalytic decomposition of MB. The ZnO/CuO/g-C_3_N_4_ (ZCG) heterostructure nanocomposite was synthesized via the co-crystallization method and employed in a photocatalytic process to eliminate MB dye from wastewater under visible light [11]. The MB degradation record was as high as 97.46% within 50 min, exhibiting superior performance to other single photocatalysts such as ZnO, CuO, and g-C_3_N_4_. This enhanced performance is attributed to the formation of heterojunction structures, promoting efficient charge transfer and reducing recombination rates [12]. Likewise, the ZnO-graphene oxide (GO) composite photocatalyst for vanillic acid (VA) degradation recorded a degradation efficiency of 99% and 35% under solar light and visible-LED, respectively [13]. This outcome is possibly caused by the adsorption of GO on ZnO. In addition, GO extends light absorption into the visible region, increasing the light harvesting effect and boosting photocatalytic activity.

In this study, carbon-based materials were composited with ZnO to improve photocatalytic activity. Activated carbon (AC) is an alternative to graphene-based materials, and it can be prepared at a low cost from any organic waste, such as agricultural wastes or weeds. Water hyacinth (Wh) is an aquatic weed deemed a nuisance in agricultural and fishing activities. Repurposing this plant as a raw material to produce AC that is composited with ZnO is a promising approach to producing low-cost, carbon-based materials. Furthermore, this strategy allows for the proper waste management of aquatic weeds, positively impacting the environment and farmers. The AC was prepared using a hydrothermal process with activating chemicals. Therefore, this study precipitated ZnO with AC to produce ZnO-AC for photocatalysis.

## 2. Materials and Methods

The zinc acetate solution was prepared by dissolving 4.39 g zinc acetate 2-hydrate (Zn(CH_3_COO)_2_·2H_2_O; KEMAUS, AR, MW 219.49) in 100 mL de-ionized (DI) water. Meanwhile, AC mixtures (1, 3, 5, and 10% of the total mass) were added to zinc acetate solution in separate beakers. The AC was prepared via high-temperature carbonization and KOH activation of dried Wh waste, as described in earlier studies [14]. The beakers were heated to 70 °C and stirred continuously for 1 h. Subsequently, the precipitation process was carried out. First, the ammonium solution was prepared by dissolving 3.16 g ammonium bicarbonate (NH_4_HCO_3_, DAEJUNG, AR, MW 79.06) in 100 mL DI water, followed by heating and stirring under similar conditions as for the zinc acetate preparation. The ammonium solution was added to the zinc acetate or mixture solutions drop-by-drop while heating and stirring to precipitate Zn(OH)_2_ or Zn(OH)_2_-AC sediment. After 1 h of precipitation, the Zn(OH)_2_ or Zn(OH)_2_-AC sediments were filtered using filter paper for 12 h, dried at 70 °C for 1 h, and ground for 3 h. Finally, the Zn(OH)_2_ and Zn(OH)_2_-AC sediments were calcined at 600 °C for 6 h and ground for 1 h to obtain ZnO or ZnO-AC.

The ZnO or ZnO-AC were utilized as photocatalysts to degrade methylene blue (MB) under ultra-violet (UV) light. The MB was dissolved in DI water at an initial concentration of 5 mg/L, stirring under dark conditions at room temperature for 30 min. Subsequently, 100 mg ZnO or ZnO-AC was added into 100 mL MB solution while stirring at room temperature in the dark. The MB solution containing ZnO or ZnO-AC was allowed to undergo adsorption–desorption stabilization for 30 min, followed by irradiation under UV light to activate photocatalysis to degrade MB molecules. The MB solution (3 mL) was sampled at different intervals, and the absorbance was measured via UV–Vis spectroscopy.

To characterize ZnO and ZnO-AC, morphology was observed using a transmission electron microscope (TEM; JEOL JEM-2100 Plus; JEOL Ltd., Tokyo, Japan). The crystalline structure was evaluated using an x-ray diffractometer (XRD; Rigaku, SmartLab, Rigaku, Japan). In addition, a Raman spectrometer (Thermo Scientific, DXR SmartRaman, Waltham, MA, USA) was used to analyze vibrational characteristics. Meanwhile, a surface area and pore-size analyzer (Quantachrome, Autosorb iQ-C-XR-XR-XR, Graz, Austria) was used to determine porosity properties based on the N_2_ adsorption–desorption method. The functional group was analyzed via the Fourier transform infrared (FTIR) spectrometer (Spectrum Two, PerkinElmer, Norwalk, CT, USA), while x-ray photoelectron spectroscopy (XPS, Kratos, Axis Ultra DLD, Kratos Analytical, Ltd., Manchester, UK) was used to monitor surface composition and chemical states.

## 3. Results and Discussion

**Figure 1:**
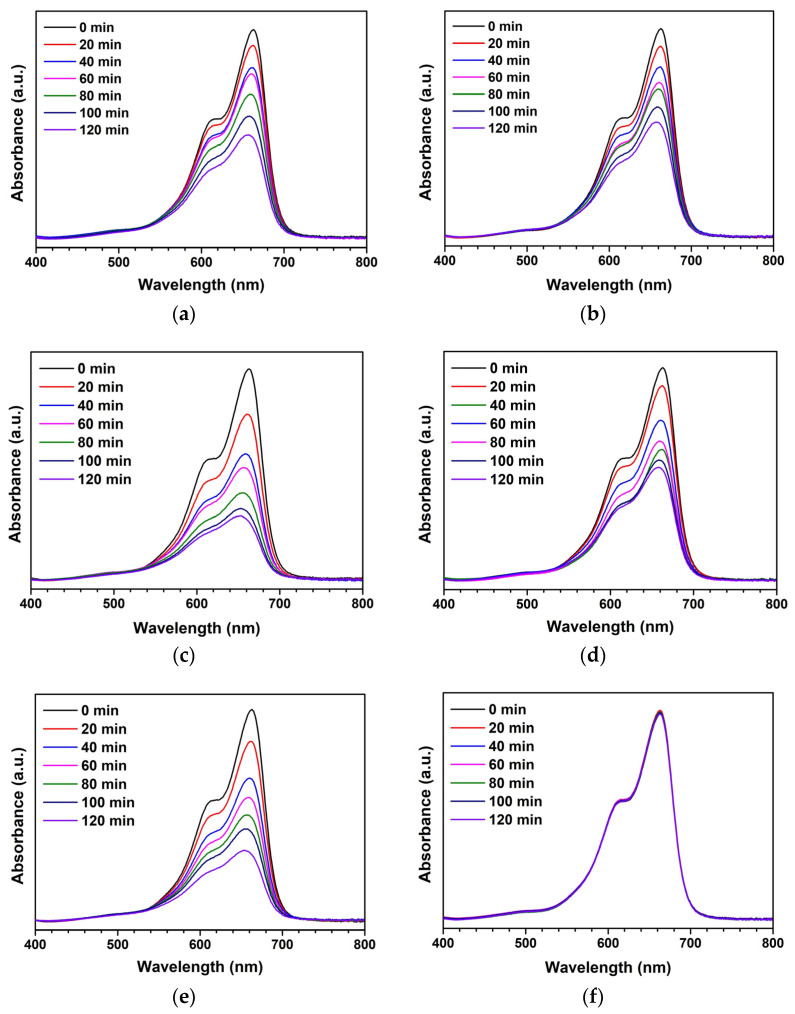
Absorbance of MB after photocatalytic activity at varying intervals of UV-irradiation time using different photocatalysts: (**a**) ZnO, (**b**) ZnO-AC1%, (**c**) ZnO-AC3%, (**d**) ZnO-AC5%, (**e**) ZnO-AC10%, and (**f**) blank.

**Figure 2:**
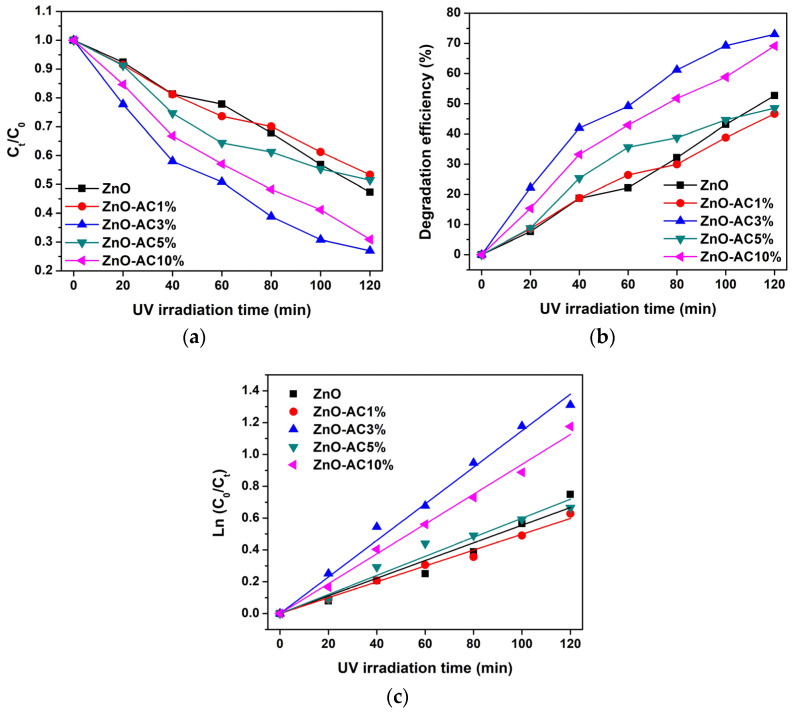
Analysis of photocatalytic degradation of MB under UV irradiation: (**a**) C_t_/C_0_ ratio, (**b**) degradation efficiency, and (**c**) degradation rate constant.

Both ZnO and ZnO-AC were utilized as UV-activated photocatalysts to degrade MB molecules. Figure 1a–e illustrates the absorbance of MB samples after photocatalysis at different UV-irradiation intervals. There was a decreasing trend in absorbance with irradiation time, indicating that MB molecule degradation in water was catalyzed by the photocatalysts used in this study. The activity of MB molecules under UV irradiation without photocatalysts (blank) is shown in Figure 1f, demonstrating no significant changes in absorbance. This finding implies that MB molecules cannot be degraded effectively without photocatalysts, thus confirming the efficacy of ZnO and ZnO-AC in this study. Additionally, the remaining concentration to initial concentration (C_t_/C_0_) ratios of MB solutions were plotted comparatively, as shown in Figure 2a, to determine the optimum photocatalyst. The degradation efficiency (DE), which corresponds to the ratio of decomposition to the initial MB molecules, was calculated using Equation (1) [15,16,17].

DE (%) = (1 − (C_t_/C_0_)) × 100% (1)

The decreasing C_t_/C_0_ trend for the ZnO-AC3% sample was the lowest compared to other conditions, which correlated with higher degradation efficiency (Figure 2b). Further quantitative analysis of photocatalytic performance was performed to determine the degradation rate constant (k_r_) (Figure 2c) using Equation (2). In addition, half-life (τ) was calculated to estimate the irradiation time required to reduce MB molecules by half of the initial concentration using Equation (3) [18,19]

ln(C_0_/C_t_) = k_r_t (2)

τ = (1/k_r_)ln(2) (3)

where t is irradiation time (min). Table 1 presents the degradation rate constant and half-life for each sample.

The highest degradation rate constant and lowest half-life were recorded for the ZnO-AC3% sample, suggesting its superiority as a photocatalyst. This result matches a previous study describing a ZnO/AC nanocomposite photocatalyst [20]. In that study, AC was prepared from *Prosopis juliflora*. This could suggest that the optimal content of AC in ZnO-AC structures is 3%. In comparison to other ZnO-based photocatalyst materials (Table 2), ZnO-AC3% can be considered for use in efficient photocatalyst applications.

**Figure 3:**
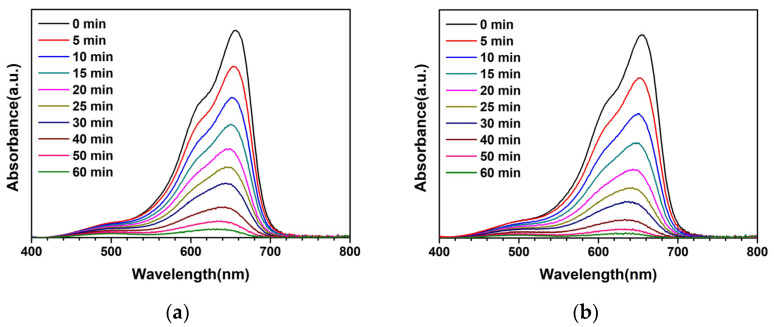

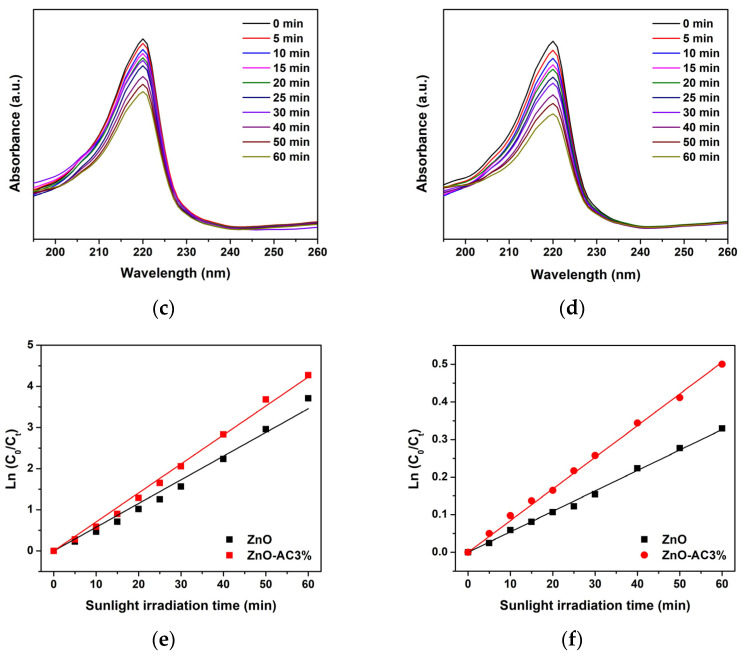
Photocatalytic degradation analysis under natural sunlight irradiation: absorbance of MB using (**a**) ZnO and (**b**) ZnO-AC3%, absorbance of CBR using (**c**) ZnO and (**d**) ZnO-AC3%, and degradation rate constant of (**e**) MB and (**f**) CBR degradation.

Thus, the ZnO-AC3% sample was further analyzed to determine its ability to degrade MB under natural sunlight [19]. Furthermore, the photocatalytic degradation of carbaryl (CBR) insecticide was also investigated by evaluating the potential of photocatalyst application in agricultural chemical degradation, demonstrating a simple way for environmental redemption. The CBR concentration was prepared at an initial concentration of 1 mg/L in DI water. Meanwhile, the average sunlight intensity was 917 W/m^2^. The absorbance of MB and CBR after photocatalytic degradation and the degradation rate constants of MB and CBR under natural sunlight with facilitation by photocatalysts are illustrated in Figure 3. The degradation rate constant was 50.61 × 10^−3^ min^−1^ for ZnO and 74.46 × 10^−3^ min^−1^ for ZnO-AC3%, indicating the superior performance of the latter in MB degradation. A similar trend was observed for CBR degradation, where ZnO-AC3% recorded a degradation rate constant of 8.43 × 10^−3^ min^−1^ and this was 5.44 × 10^−3^ min^−1^ for ZnO. Therefore, ZnO-AC3% has been proven effective as a photocatalyst for MB and CBR degradation, offering a potential application in contaminated areas under natural sunlight.

**Figure 4:**
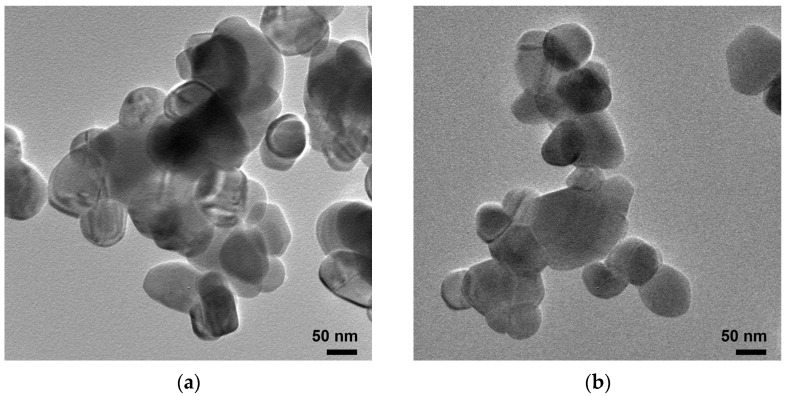
TEM images of (**a**) ZnO and (**b**) ZnO-AC nanostructures.

The ZnO-AC3% was also utilized as a representative ZnO-AC structure for comparison with pristine ZnO for TEM analysis (Figure 4). The images revealed that both photocatalysts had spherical-like nanostructures and comparable sizes, suggesting no discernible impact of AC on the morphological structure of ZnO. Nonetheless, other characteristics of AC structures, such as flake-, sheet-, or plate-like structures, were not identified due to the limited sample availability.

**Figure 5:**
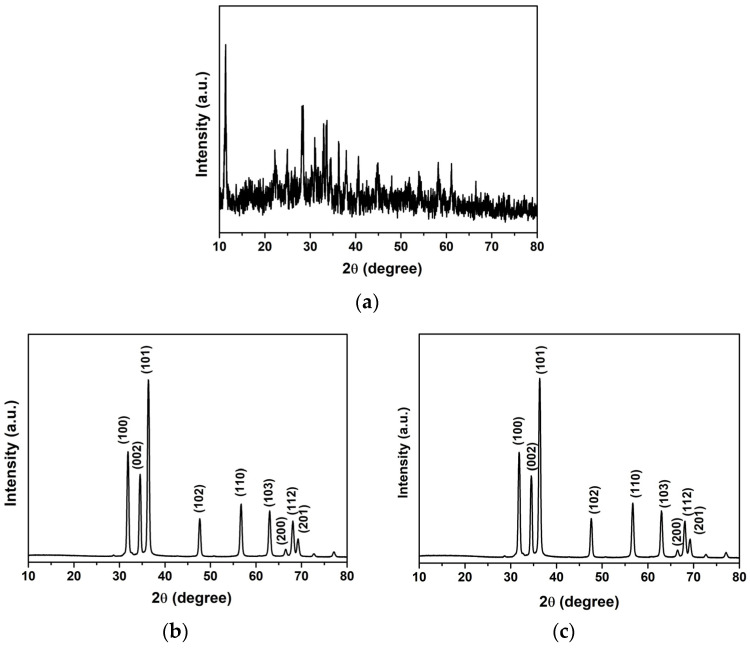
XRD patterns of (**a**) AC, (**b**) ZnO, and (**c**) ZnO-AC.

The crystalline structures of AC, ZnO, and ZnO-AC were also evaluated through analysis of their XRD patterns (Figure 5). Carbon-related peaks were unclearly observed for AC. This indicated an amorphous structure for the AC sample. Regarding the unidentified peaks, there should be several mineral components in AC due to the use of biomass as a raw material. For ZnO and ZnO-AC, consistent diffraction peaks were evident with distinct patterns observed at 2θ of 31.8°, 34.6°, 36.4°, 47.6°, 56.9°, 63.0°, 66.6°, 68.1°, and 69.3° that correspond to the (100), (002), (101), (102), (110), (103), (200), (112), and (201) diffraction planes, respectively. These characteristic planes strongly suggested the presence of a ZnO-hexagonal-wurtzite structure in both photocatalysts, which aligned with the JCPDs no. 36-1451 standard.

**Figure 6:**
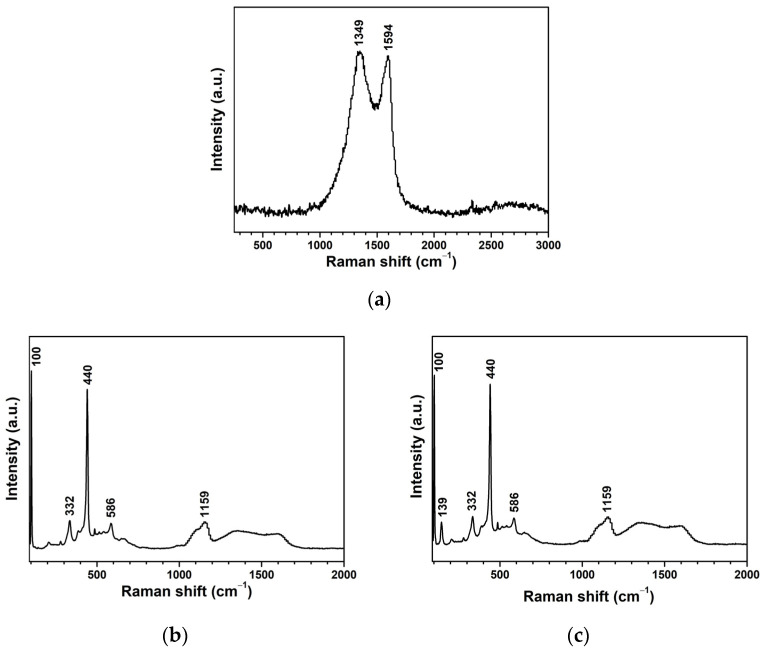
Raman spectroscopy analysis of (**a**) AC, (**b**) ZnO, and (**c**) ZnO-AC.

The findings from the Raman spectroscopy are presented in Figure 6. Figure 6a presented disordered and graphitic peaks of AC at 1349 and 1594 cm^−1^, respectively, which implied a high order of the amorphous carbon structure. The Raman shift analysis of ZnO (Figure 6b) and ZnO-AC (Figure 6c) exhibited consistent peaks at 332, 440, 586, and 1159 cm^−1^ [25,26,27,28]. The peak at 332 cm^−1^ was assigned to the E_2_(high)–E_2_(low) mode, while the strong peak at 440 cm^−1^ corresponded to the E_2_(high) modes due to the oxygen vibration in the ZnO matrix. Meanwhile, the two peaks at 586 and 1159 cm^−1^ correspond to the E_1_(LO) and 2E_1_(LO) modes representing the multi-phonon process. These outcomes indicate the intrinsic nature of ZnO hexagonal wurtzite structures in ZnO and ZnO-AC samples, consistent with the XRD results. However, the peaks of AC were not observed in the ZnO-AC samples, which might be due to the low AC content in the ZnO-AC sample.

**Figure 7:**
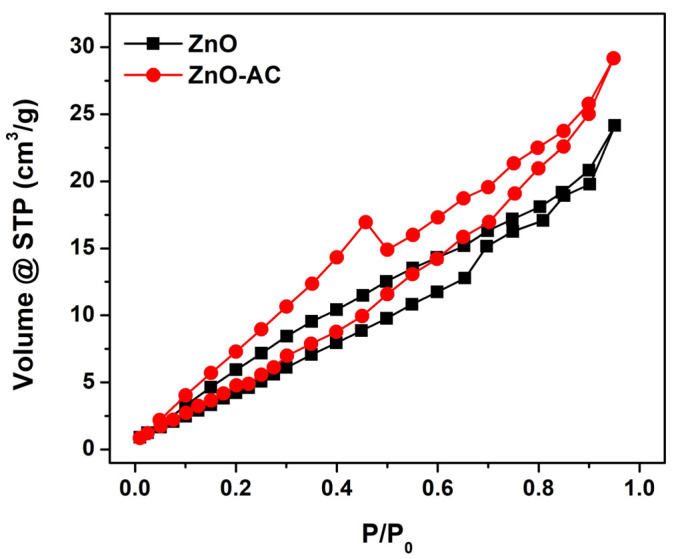
N_2_ adsorption–desorption isotherms of ZnO and ZnO-AC.

The physicochemical properties of ZnO and ZnO-AC samples were investigated using the N_2_ adsorption–desorption method. Figure 7 demonstrates that the N_2_ adsorption–desorption isotherm of ZnO and ZnO-AC features type H3 hysteresis due to the single-layer adsorption stage, which could be attributed to the ZnO solid structure. Moreover, the adsorption–desorption isotherm indicates that both photocatalysts have meso- or macro-porous structures. The Barrett–Joyner–Halenda (BJH) adsorption analysis was conducted to determine ZnO and ZnO-AC porosities (Table 3). ZnO and ZnO-AC each demonstrated a mesoporous structure with pore diameters of 6.55 and 3.83 nm, respectively. The smaller pore diameter of ZnO-AC resulted in larger pore volume and higher BET surface area (S_BET_) than those of ZnO, as calculated using the multipoint Brunauer–Emmett–Telle (BET) method.

**Figure 8:**
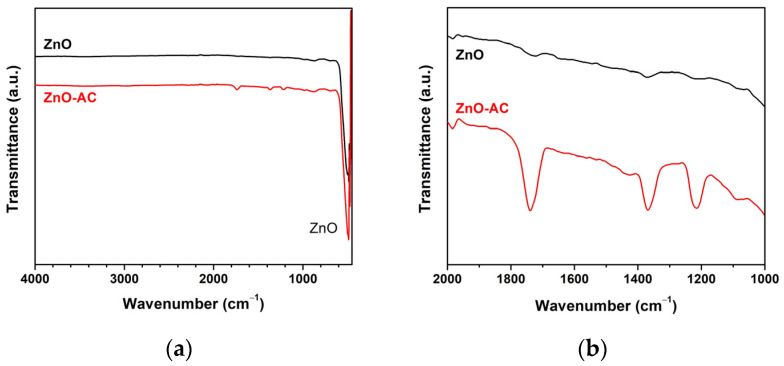
FTIR analysis of ZnO and ZnO-AC using (**a**) full scanning, and (**b**) functional group scanning.

The FTIR spectra revealed a major peak at 490 cm^−1^, indicating the Zn-O vibration of the ZnO matrix in the structural composition of both photocatalysts (Figure 8a) and the prevalence of ZnO structures in these examined samples. Meanwhile, the functional group analysis demonstrated that the wavenumber in the ZnO-AC sample ranged between 2000 and 1000 cm^−1^ (Figure 8b). The distinct vibrations at wavenumbers of 1738, 1367, and 1214 cm^−1^ represented characteristic peaks associated with C=O stretching, methyl groups, and C-H bending, respectively [29,30]. These findings are particularly significant as they suggest the potential adsorption of carbon on the ZnO surface, a phenomenon that can likely be attributed to the presence of AC in the ZnO-AC composite.

**Figure 9:**
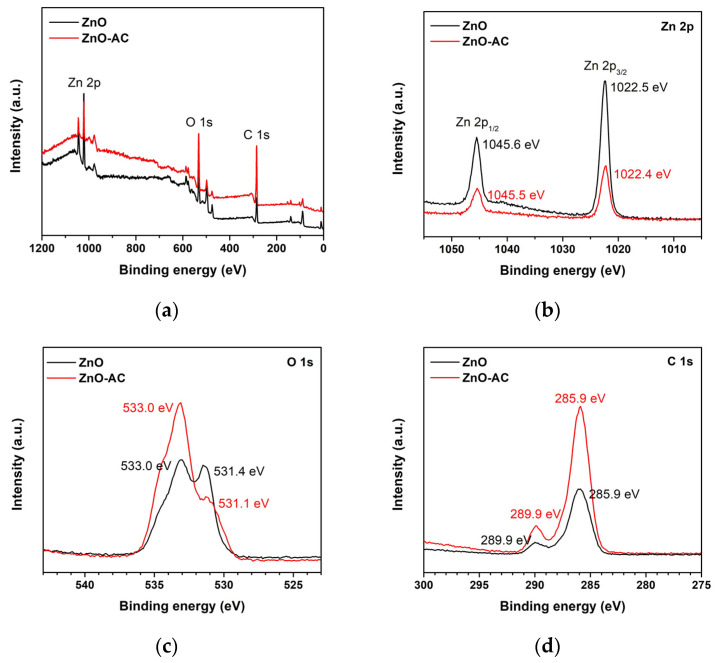
XPS analysis of ZnO and ZnO-AC: (**a**) full scan, (**b**) Zn 2p, (**c**) O 1s, and (**d**) C 1s.

The binding energy (BE) of each sample was investigated using XPS to evaluate the ZnO-AC surface (Figure 9). In Figure 9a, the survey XPS spectra of ZnO and ZnO-AC identified chemical elements Zn, O, and C, reflecting comparable chemical compositions for the samples. The high-resolution Zn 2p core level in Figure 9b illustrates the distinct Zn 2p_3/2_ and Zn 2p_1/2_ peaks of ZnO exhibited at BEs of 1022.5 and 1045.6 eV, respectively, signifying the Zn^2+^ oxidation state [31]. Notably, there were no differences in these peaks between ZnO and ZnO-AC. The O 1s spectra of ZnO depicted peaks at BEs of 531.4 and 533.0 eV (Figure 9c), corresponding to O^2−^ ions in the metal oxide and compound groups, respectively [32]. The O^2−^ ions were associated with the bonding of Zn^2+^ ions in the ZnO structure, whereas the compound groups indicated the chemisorption on the ZnO surface responsible for the oxygenated carbon components in this case [33]. Meanwhile, a decrease in O^2−^ ions and an increase in the compound group were evident in the O 1s spectra of ZnO-AC, suggesting a stronger chemisorption effect of AC on ZnO structures. Figure 9d shows the C 1s spectra with peaks at BEs of 285.9 and 289.9 eV for ZnO, corresponding to C-O and C=O groups, respectively [11]. This might be due to residual levels of the starting ammonium bicarbonate. However, the relatively elevated intensity of these peaks in the ZnO-AC sample confirms the presence of AC on the ZnO surface. This detailed XPS analysis provides valuable insights into the surface compounds of ZnO-AC, which agree with the FTIR results.

**Figure 10:**
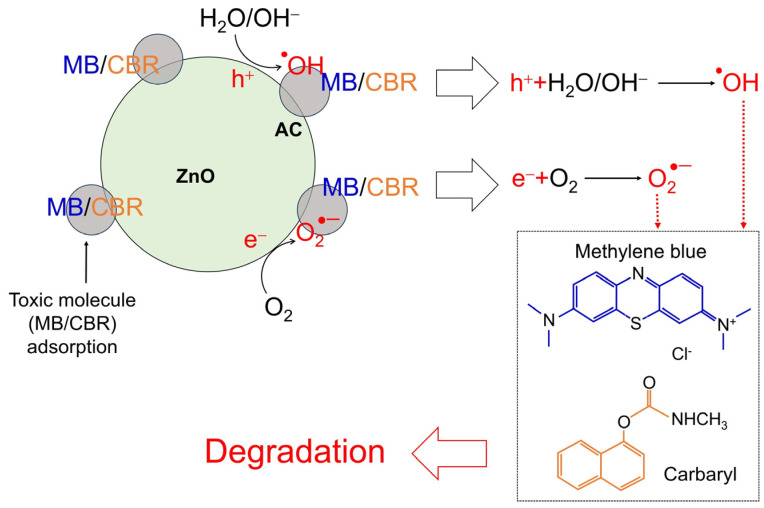
The mechanism of contaminant degradation using a ZnO-AC photocatalyst.

The function of the adsorbing porous material of AC in caping surrounding toxic molecules near the ZnO-AC surface for improving photocatalytic mechanisms is assumed, as illustrated in Figure 10. In conventional photocatalysis, after electron–hole pairs are generated due to the incident light irradiation, there is a reaction in which dissolved oxygen (O_2_) forms superoxide anions (O_2_^•−^). Simultaneously, holes react with water (H_2_O) or hydroxyl groups (OH^−^), forming hydroxyl radicals (^•^OH). These reactive oxygen species (ROS) are continuously reacting with toxic molecules. In the case of ZnO-AC, toxic molecules were adsorbed by AC at a rapid rate and they reacted with ROS in the photocatalytic activity process for mineralization [34,35]. Thus, the presence of porous AC on ZnO surfaces plays a key role in the increasing photocatalytic activity rate. This mechanism can increase reaction density in the photocatalytic process and boost photocatalytic performance.

This study has briefly shown that aquatic weeds are of benefit as carbon-rich sources for transforming into carbon powder materials. The transforming process of Wh not only contributes to aquatic weed management but also serves as a viable carbon storage strategy. Employing a chemical activation process, carbon powder materials are activated to enhance surface properties, resulting in AC. This AC is then utilized to modify the ZnO photocatalyst, demonstrating the enhancement of photocatalysis under sunlight irradiation to degrade toxic molecules, including methylene blue and carbaryl. Thus, using water hyacinth as a carbon source for the transformation of AC can alternatively solve the problems of aquatic weed management and carbon storage strategies, and the application of AC to ZnO-AC photocatalysts can enhance photocatalysis.

## 4. Conclusions

Water hyacinth (Wh) is an aquatic weed considered a nuisance in agricultural and fishing activities. Therefore, this study proposed repurposing this plant into activated carbon (AC). First, the ZnO-AC was precipitated and applied as a photocatalyst for degrading methylene blue. For the preliminary photocatalytic test of methylene blue (MB) degradation, ZnO-AC recorded the highest degradation rate constant of 11.49 × 10^−3^ min^−1^, which was double the value of pristine ZnO (5.55 × 10^−3^ min^−1^). Furthermore, the photocatalytic degradation of MB and CBR under sunlight irradiation by ZnO-AC demonstrated degradation rate constants of 74.46 × 10^−3^ min^−1^ and 8.43 × 10^−3^ min^−1^, respectively. To investigate the properties of ZnO-AC, several techniques were performed. ZnO-AC and ZnO exhibited similar results in morphology, crystalline structure, and Raman characteristics. However, ZnO-AC presented smaller pore diameters than those of ZnO, which enlarged the relevant surface area, and the presence of carbon-related groups implied the presence of AC on ZnO-AC surfaces. This can be attributed to the presence of AC on the ZnO surface, increasing the capture rate of surrounding toxic molecules and elevating the reaction density. This enhancement is thought to promote the degradation of toxic molecules. Therefore, using Wh as a carbon source for the transformation of AC can alternatively solve the problems of aquatic weed management and carbon storage strategies, and the application of AC to ZnO-AC photocatalysts can enhance photocatalysis.

## Figures and Tables

**Table 1:** Analytical parameters of MB degradation under UV irradiation.

Sample	k_r_ (10^−3^ min^−1^)	τ (min)	R^2^
ZnO	5.55	125	0.9814
ZnO-AC1%	4.98	139	0.9960
ZnO-AC3%	11.49	60	0.9966
ZnO-AC5%	5.99	116	0.9884
ZnO-AC10%	9.38	74	0.9975

**Table 2:** Previously reported analytical degradation rate constant of MB degradation.

Sample	MB Concentration	Photocatalyst Dosage in MB Solution	Light Source	k_r_ (10^−3^ min^−1^)	Ref.
Ti-ZnO	5 mg/L	0.1 g/100 mL	UV	2.54	[5]
GQDs-ZnO	10 mg/L	0.2 g/100 mL	UV	3.79	[21]
rGO@ZnO	15 mg/L	20 mg/100 mL	Sunlight	5.03	[22]
N-ZnO	10 mg/L	0.1 g/100 mL	Sunlight	19.6	[23]
Cu-ZnO	10 mg/L	25 mg/100 mL	UV	25.4	[24]
ZnO-AC3%	5 mg/L	0.1 g/100 mL	UV	11.49	This work

GQDs: graphene quantum dots; Ti: titanium; rGO: reduced graphene oxide; N: nitrogen; Cu: copper.

**Table 3:** BJH porosities and BET surface areas of ZnO and ZnO-AC.

Sample	Pore Diameter (nm)	Pore Volume (cm^3^/g)	S_BET_ (m^2^/g)
ZnO	6.55	3.44 × 10^−2^	27.08
ZnO-AC	3.83	4.40 × 10^−2^	28.99

## Data Availability

Data are contained within the article.
